# Psychiatrists and COVID-19: what is our role during this unprecedented time?

**DOI:** 10.1017/ipm.2020.95

**Published:** 2020-08-19

**Authors:** M. Scriven, E. Geary, B. D. Kelly

**Affiliations:** 1 Department of Psychiatry, North Lee Mental Health Services, Mercy University Hospital, Cork, Ireland; 2 Department of Psychiatry, South Lee Mental Health Services, Cork University Hospital, Cork, Ireland; 3 Department of Psychiatry, Trinity College Dublin, Trinity Centre for Health Sciences, Tallaght University Hospital, Dublin, Ireland

**Keywords:** COVID-19, mental health services, pandemic

## Abstract

The declaration of a COVID-19 (Severe Acute Respiratory Syndrome – CoronaVirus2) pandemic by the World Health Organization in March 2020 has vastly changed the landscape in which mental health services function. Consideration is required to adapt services during this unusual time, ensuring continued provision of care for current patients, availability of care for patients with new-onset mental health difficulties and delivery of evidence-based support for healthcare professionals working with affected patients. Lessons can be learned from research carried out during the severe acute respiratory syndrome, Middle East respiratory syndrome and Ebola epidemics to ensure the delivery of efficient and effective mental health services both now and into the future.

## Introduction

In March 2020, the World Health Organisation (WHO) declared that the current outbreak of COVID-19 (SARS-CoV2) to be a pandemic (Bedford *et al.* 2020). Pandemics are characterised by the presence of a novel infectious disease that affects a large geographic area, with high transmission rates and minimal immunity (Morens *et al.* 2009). The consequences of the COVID-19 pandemic are enormous, with mortality estimated to be between 1% and 7% of those diagnosed, depending on the testing strategy chosen by each country (Vincent & Taccone, 2020). As of May 2020, over 55% of deaths have occurred in Europe, but the full medical, economic, educational and social repercussions of the outbreak around the world have yet to be established (WHO, 2020).

It has been reported that the pandemic will increase psychiatric morbidity in both the short term and the long term (Holmes *et al.* 2020; Goldman, 2000), with a suggestion that this morbidity will last longer and peak later than the pandemic itself (Gunnell *et al.* 2020). The precise nature and extent of the mental health challenges are, however, still unknown owing to the acute and novel nature of COVID-19 (Xiang *et al.* 2020a). To date, the efforts of the scientific and medical communities have been focused on the epidemiology, clinical presentations and emergency management of COVID-19, with limited focus on the mental health aspect of the pandemic (Shah *et al.* 2020b).

As a result, the precise roles that psychiatrists will play in ameliorating the broader consequences of the pandemic are not yet fully clear. Many different groups are at risk of the psychological effects of COVID-19 including patients who contract the infection, bereaved family members, healthcare professionals providing care, and patients with pre-existing psychiatric diagnoses (Pfefferbaum & North, 2020). Several risk factors for developing or exacerbating psychiatric illness are especially relevant during a pandemic, including lack of social contact, physical illness, domestic violence and financial difficulties (Holmes *et al.* 2020). Many of these risk factors are also relevant to rates of suicide (Gunnell *et al.* 2020).

There are, as a result, many challenges for psychiatrists during this unprecedented time, with mental healthcare rendered more complex as infection control measures such as social distancing and personal protective equipment commonly act as barriers to effective communication. In this situation, it is necessary to adjust services to meet existing patients’ new needs, adapt services to assess and treat new patients, and provide evidence-based support to our medical colleagues. It is important that these efforts are underpinned by close study of both pre-existing research from past epidemics, such as Middle East respiratory syndrome (MERS) in 2015 and severe acute respiratory syndrome (SARS) in 2003, and emerging research from the current pandemic. However, the differences between COVID-19, SARS and MERS need to be noted when considering previous research. The mortality rate of COVID-19 (2.3%) is lower than both SARS (9.5%) and MERS (34.4%), and COVID-19 generally spreads in the community due to its less severe clinical picture, compared to the nosocomial spread of both SARS and MERS (Petrosillo *et al.* 2020). The lessons learned from these previous epidemics should inform future planning with an acknowledgement of the likely different consequences.

It is through this lens that plans for future mental health service provision should be developed. This article aims to describe research findings from past epidemics and suggest actions that can be taken to best meet the needs of patients, healthcare professionals and the general public now.

## COVID-19 and people with pre-existing mental illness

The outbreak of COVID-19 is concerning for patients with pre-existing psychiatric diagnoses owing to disruptions to community services, decreased social contact, worsening of pre-existing symptoms (Holmes *et al.* 2020) and the possibility of redeployment of healthcare staff (HSE, 2020). Risk of contracting the virus itself is another significant concern. Patients with chronic and enduring psychiatric illness are more vulnerable to medical illness than members of the general public, with particularly increased risk of diabetes, respiratory and liver disease (Sokal *et al.* 2004). This might also apply to their susceptibility to COVID-19 (Yao *et al.* 2020).

These wider health consequences of contracting COVID-19 are starting to become apparent with emerging research reporting kidney and liver injury as well as thrombotic events (Rismanbaf & Zarei, 2020, Klok *et al.* 2020). These findings are particularly worrying for patients with psychiatric illness due to their increased risks of both underlying medical conditions and thrombosis, if prescribed anti-psychotics (Thomassen *et al.* 2001). Inpatients in psychiatric units are thought to be especially vulnerable to contracting COVID-19, compared to medical inpatients, as a result of the layout of inpatient beds and shared dining and social spaces (Xiang *et al.* 2020b). Certain countries have suggested specific inpatient units for patients with psychiatric diagnoses and COVID-19 because the suggested guidelines are not always appropriate for patients with psychiatric diagnoses (Chevance *et al.* 2020).

Some countries have also published comprehensive protocols for the use of phone contact, conducting outpatient appointments and organising inpatient admissions in psychiatric services during the pandemic (Starace & Ferrera 2020; Xiang *et al.* 2020b). The imperative to continue providing multidisciplinary care has been noted (Kelly 2020). Investing in online interventions for patients who require support during this time has been suggested as one method for maintaining services while minimising face-to-face contact between patients and healthcare professionals (Holmes *et al.* 2020). Patients’ increased access to the Internet and ownership of smartphones during the current pandemic, compared to the SARS epidemic, has been noted (Liu *et al.* 2020).

Despite these measures and advances, however, there are still several challenges associated with remote treatment (Gunnell *et al.* 2020) including persisting issues about access to online interventions (Li *et al.* 2020). Significant investment is likely needed to improve availability of Internet throughout Ireland, with nearly 60% of the Irish public reporting dissatisfaction in the quality of their Internet (Burke-Kennedy 2019). The provision of online resources also needs to consider previous reports of social media having a negative impact on patients with depressive, anxiety and psychotic disorders (Primack *et al.* 2017). All services provided to existing patients need to simultaneously meet patients’ psychiatric needs and reiterate the importance of public health recommendations. Additionally, there is a need for psychiatrists to engage in prospective, high-quality research in relation to the broader consequences of this pandemic, with particular emphasis on mental health (Holmes *et al.* 2020).

## Psychiatry and people who recover from COVID-19

To date, over 23,000 cases of COVID-19 have been diagnosed in Ireland (Department of Health, 2020). Although it is not yet clear what proportion of these patients will develop a psychiatric illness in need of specialist care, there are some indicators from previous epidemics. Past research has reported quite strong links between acute infections and psychiatric illness, with an established association between influenza and psychosis (Kepińska *et al.* 2020) and a correlation between disease severity in SARS and neuropsychiatric symptoms (Sheng *et al.* 2005).

The SARS outbreak has been described as a mental health catastrophe for survivors, owing to the prolonged duration of threat, the infection’s unpredictable nature and the resulting social isolation. Contracting and surviving SARS resulted in an increased risk of both depressive and anxiety disorders (Mak *et al.* 2009). The psychiatric morbidity of SARS also appears to be long-lasting, with 64% of SARS survivors scoring above the threshold of the General Health Questionnaire (GHQ12) at 1-year follow-up (Lee *et al.* 2007). There were also significant rates of chronic fatigue and psychiatric disorders at 3-year follow-up (Wing & Leung, 2012). These findings underline the importance of identifying, articulating and normalising the fears and stresses experienced by patients with these illnesses and placing particular emphasis on explaining anxiety as an appropriate response to an overwhelming event such as SARS (Maunder *et al.* 2003).

Liaison psychiatry has several potential roles to play during this time, including aiding in the management of delirium, dysregulation and decompensation of patient’s mental state secondary to contracting COVID-19, managing drug interactions between psychotropic drugs and medications used in the treatment of the virus and advocating for patients with pre-morbid psychiatric diagnoses (Shalev & Shapiro, 2020; Vieta *et al.* 2020).

In keeping with current models of mental healthcare, a stepped approach should be taken when managing the psychiatric sequelae of COVID-19. Care in general practice is the first step, involving psychological therapy or medication (if indicated) as the first-line treatment, depending on the clinical situation. Referral to specialist mental health services might be required if symptoms fail to improve or if severe, acute symptoms predominate, as is often seen in emergency departments (HSE Mental Health Division, 2017). Particular attention needs to be given to older adults who are most affected by COVID-19 (Yang *et al.* 2020). Specific training for psychiatrists to prevent and detect post-traumatic stress disorder has been suggested due to its prevalence in Wuhan, China (Chevance *et al.* 2020).

## COVID-19 and healthcare colleagues

Past experience suggests that COVID-19 will likely result in significant psychiatric morbidity among healthcare professionals, based on the documented sequelae of the SARS, MERS and Ebola epidemics (Shah *et al.* 2020a). Such morbidity affects not only professionals’ own mental health but also their ability to provide effective care (Kang *et al.* 2020). Following the SARS outbreak, healthcare professionals in direct contact with infected patients had significantly higher levels of stress, anxiety and depression at 1-year follow-up compared to healthcare workers who were not in direct contact with affected patients (McAlonan *et al.* 2007).

The concept of resilience is important in this context. The development of psychiatric symptoms in the aftermath of treating patients with SARS was significantly associated with a history of mental illness and inversely associated with years working in healthcare. The rates, however, were similar to incidence rates in the community, suggesting a certain amount of resilience among healthcare workers, protecting their mental health (Lancee *et al.* 2008).

It has been suggested that developing a culture of resilience at an organisation level reduces the stress levels of individual staff members (Maunder *et al.* 2008). The provision of both practical and emotional support is recommended to increase such resilience, especially for individuals with a history of mental illness (Lancee *et al.* 2008). Investing in telepsychiatry has also been suggested to help avoid burnout in healthcare professionals (Shah *et al.* 2020a). Online interventions have also been discussed, although barriers include a lack of time and energy to engage with these supports (Li *et al.* 2020). Crisis interventions for healthcare professionals were suggested by the National Health Committee in China in early 2020, informed by the SARS outbreak of 2003 (Xiang *et al.* 2020a).

There is also a risk of ‘moral injury’ when healthcare professionals have to make difficult treatment decisions with limited resources and high volumes of patients. This can result in negative cognitions and emotions which can, in turn, contribute to psychological and psychiatric symptoms (Greenberg *et al.* 2020). In clinical situations where healthcare professionals act in a manner that is not in keeping with their moral code, peer support rather than psychological debriefing should be prioritised (Brooks *et al.* 2018; Greenberg *et al.* 2020).

## COVID-19, psychiatry and the general public

Although most members of the general public will not meet the threshold for specialist psychiatric input, there are several ways in which psychiatry can assist the public during and after COVID-19. These include informing digital interventions to address psychological distress (Gunnell *et al.* 2020), liaising with other agencies that provide support to people with low-grade symptoms and creating educational resources for general practitioners and other clinical colleagues who are addressing pandemic-specific psychological symptoms.

The MERS outbreak in Korea resulted in widespread anxiety concerning the lack of treatment, vaccination and the need for people to self-isolate (Jeong *et al.* 2016). Mandatory quarantine results in psychological effects that are not insignificant (Rubin & Wessely, 2020), owing, not least, to the uncertainty and shifting parameters that characterise the early stages of most major public health emergencies (Johal, 2009). Increased rates of suicide were found in the older adult population in Hong Kong during the SARS outbreak, with fear of burdening families, social isolation and anxiety associated with some of these deaths (Yip *et al.* 2010). The normalisation of such fears can ameliorate the psychological impact of quarantine and other public health measures (Johal, 2009). Engaging with reputable sources of information also assists with maintaining a positive outlook (Shah *et al.* 2020b).

The decrease in organisations providing supportive counselling is likely to have a knock-on effect for the psychiatric services, with some organisations moving from their usual services to the provision of online information and telephone support between March and July 2020 (Walsh, 2020).

The overwhelming amount of misinformation readily available online is another concern for the public, as high-speed Internet allows the dissemination of information – both reliable and unreliable – to large numbers of people. Various media channels are now being used to question the legitimacy of conspiracy theories that can undermine public health efforts (Ignatius, 2020). The WHO has described the rapid spread of misinformation about COVID-19 as an ‘infodemic’ (Limaye *et al.* 2020).

Although it is tempting simply to dismiss conspiracy theories, we should not underestimate their potential to cause harm. Decreased uptake of the mumps, measles and rubella vaccine owing to various unfounded concerns are just one example of this (Andrade, 2020). There is a need for all healthcare professionals to both provide accurate information and dismiss inaccurate information, especially among patients who are especially vulnerable to external influences.

The likelihood of an economic downturn as a consequence of COVID-19 also needs to be considered, with the previous recession in Ireland resulting in increased rates of suicide in males and increased rates of self-harm in both males and females (Corcoran *et al.* 2015).

## Conclusion

The consequences of COVID-19 are likely to be significant and long-lasting across all medical specialities, including psychiatry. Considered and detailed planning is required to meet the mental health needs of current patients and other people affected by COVID-19, including healthcare professionals and the general public, especially if austerity measures follow the pandemic. The current literature suggests that people with pre-existing psychiatric illness need to be monitored for an exacerbation of symptoms, that patients who recover from COVID-19 are at increased risk of psychiatric illness, and that psychological support benefits healthcare professionals caring for those who contract the virus during these difficult, unprecedented times.
